# Exertional heat stress and intestinal barrier injury: Does chronic disease add fuel to the fire?

**DOI:** 10.1113/EP092759

**Published:** 2025-03-31

**Authors:** Oliver R. Gibson, Zachary J. McKenna

**Affiliations:** ^1^ Department of Sport, Health and Exercise Sciences Brunel University of London Uxbridge UK; ^2^ Centre for Physical Activity in Health and Disease (CPAHD) Brunel University of London Uxbridge UK; ^3^ Institute for Exercise and Environmental Medicine Texas Health Presbyterian Hospital Dallas Dallas Texas USA; ^4^ Department of Internal Medicine University of Texas Southwestern Medical Center Dallas Texas USA

**Keywords:** diabetes, gut injury, hypertension, hyperthermia

1

In this issue of *Experimental Physiology* Lee and colleagues sought to examine whether older adults with type 2 diabetes or hypertension, compared to age‐matched adults without chronic disease, exhibit greater intestinal damage, microbial translocation, and inflammation during exertional heat stress replicating occupational work (Lee et al., 2025). The global demographic shift towards an ageing population, coupled with declining physical activity levels and an associated rise in non‐communicable diseases, poses a significant threat to public health and healthcare systems worldwide. Adding to these challenges is an existential crisis in the form of climate change, characterized by rising average surface temperatures and a growing frequency and intensity of extreme heat events, such as heatwaves. These conditions pose significant threats to human physiological function, impacting all aspects of daily life, including work and occupational activities. In light of this, the study from Lee et al. (2025) is timely.

Recent work has identified that healthy older adults are likely at greater risk of gut injury, a contributory factor in heat illness, relative to healthy young adults (Foster et al., 2023; McKenna et al., 2024). Lee et al. (2025) now extend our understanding by demonstrating that following a prolonged occupationally relevant exertional stress test, older men experienced greater intestinal injury, microbial translocation and inflammation when exercising in hot versus temperate conditions. In addition to ageing, the presence of type 2 diabetes and hypertension are factors that exacerbate an individual's heat illness risk. Lee et al. (2025) identify that even when these disease states are well controlled, exercise tolerance in the heat is impaired relative to healthy age‐matched peers. In addition to impaired tolerance to the exertional heat stress testing, the cohort with type 2 diabetes experienced greater enterocyte damage and microbial translocation (Lee et al., 2025). Together these outcomes demonstrate that completing physical activity at an intensity congruous with many occupational contexts places the older adult at risk of gastrointestinal injury, and perhaps heat illnesses, when hyperthermia occurs.

The findings of Lee et al. (2025) provide important novel insights that set a strong foundation for future research. However, there are a few considerations that should be highlighted. Most notably, the studied participants had well‐controlled hypertension or type 2 diabetes. This is important for at least two reasons. First, it is unclear how responses in individuals with controlled chronic diseases (e.g., hypertension and type 2 diabetes) compare to those with more severe disease progression under heat stress. For example, mechanisms that contribute to intestinal barrier dysfunction under these conditions include elevated basal inflammation, decreased microbial neutralization and microbial dysfunction at rest under heat stress, yet it is unclear to what extent these issues persist when the disease is controlled (König et al., 2016). Nonetheless, the fact that the authors noted greater elevations in enterocyte damage and microbial translocation in the cohort with controlled type 2 diabetes indicates that these issues may be even more greatly exacerbated in those with uncontrolled or more severe chronic health conditions. Second, and equally important are the impacts of the various medications that are commonly used to control these diseases, which as of now are not well understood. For example, participants in the study reported taking a range of medications including calcium channel blockers, diuretics, metformin, dipeptidyl peptidase‐4 inhibitors and sodium–glucose transport protein 2 inhibitors, among others. Of particular interest to the gastrointestinal system is the recent use of medications that act on the glucagon‐like peptide‐1 pathway. It is not entirely clear what effect, if any, these medications have on the responses to exertional heat stress. Perhaps these medications strengthen the intestinal barrier, or perhaps some of these medications make the gut more vulnerable to heat injury.

Recent, related work in *Experimental Physiology* has indicated that biomarkers associated with hyperthermia‐derived intestinal injury may be subject to substantial inter‐individual responses to equivalent core temperature (Gibson et al., 2025; McKenna et al., 2024), and that consideration of a responder/non‐responder paradigm may be relevant (Gibson et al., 2025). Lee et al. (2025) also demonstrate substantial inter‐individual differences in the magnitude of change in their examined biomarkers in support of this proposal. At the current time, experimental designs appropriate to confirm a responder/non‐responder construct have yet to be implemented and primary mechanisms associated with heat illness remain equivocal, highlighting the need for further work in this field.

Collectively, Lee et al. (2025) demonstrate that chronic health conditions (type 2 diabetes, and hypertension) impair exercise tolerance in the heat in older adults, with type 2 diabetics also at increased risk of gut‐derived heat injury during exertional heat stress. The mechanisms associated with declines in the gut's ability to withstand perturbations elicited during hyperthermia remain incompletely understood and warrant careful examination, particularly in older adults and those burdened by disease. Future work should also consider an exploration of medications that are commonly used to treat common non‐communicable diseases to determine how they impact both the thermoregulatory and downstream responses (i.e., biochemical) to heat stress. Likewise, an examination of those with uncontrolled clinical conditions is needed to discern if the responses are related to disease severity. Finally, studies identifying the inter‐individual responses to exertional heat stress are needed to discern why certain individuals respond while others do not, which will help us better understand the role of the gut in the heat injury paradigm.

## AUTHOR CONTRIBUTIONS

Both authors have approved the final version of the manuscript and agree to be accountable for all aspects of the work in ensuring that questions related to the accuracy or integrity of any part of the work are appropriately investigated and resolved. All persons designated as authors qualify for authorship, and all those who qualify for authorship are listed.

## CONFLICT OF INTEREST

None declared.

## FUNDING INFORMATION

None.
